# IFNλ: balancing the light and dark side in pulmonary infection

**DOI:** 10.1128/mbio.02850-22

**Published:** 2023-06-06

**Authors:** Danielle Antos, John F. Alcorn

**Affiliations:** 1 Division of Pulmonary Medicine, Department of Pediatrics, UPMC Children’s Hospital of Pittsburgh, Pittsburgh, Pennsylvania, USA; 2 Department of Immunology, University of Pittsburgh, Pittsburgh, Pennsylvania, USA; Ohio State University, Columbus, Ohio, USA; The Ohio State University Wexman Medical Center, Columbus, Ohio, USA

**Keywords:** lung, virus, bacteria, interferon, immunity

## Abstract

Interferon (IFN) represents a well-known component of antiviral immunity that has been studied extensively for its mechanisms of action and therapeutic potential when antiviral treatment options are limited. Specifically in the respiratory tract, IFNs are induced directly on viral recognition to limit the spread and transmission of the virus. Recent focus has been on the IFNλ family, which has become an exciting focus in recent years for its potent antiviral and anti-inflammatory activities against viruses infecting barrier sites, including the respiratory tract. However, insights into the interplay between IFNλs and other pulmonary infections are more limited and suggest a more complex role, potentially detrimental, than what was seen during viral infections. Here, we review the role of IFNλs in pulmonary infections, including viral, bacterial, fungal, and multi-pathogen super-infections, and how this may impact future work in the field.

## INTRODUCTION

Interferons (IFNs) are central to the innate immune response against viruses. There are three major classes of IFNs, but type I IFNs and the more recently discovered type III IFNs are induced directly on viral recognition (1). Type I IFNs are primarily composed of IFNα and IFNβ, with additional lesser studied subtypes including IFNε and IFNω (2), and type III IFNs include IFNλ1–3, also named as interleukin (IL)-29, IL-28A/B, and IFNλ4 (3 - 5). While initially considered to be functionally redundant, a large body of work has emerged outlining the non-redundant roles of these IFNs during infection. IFN receptors are heterodimeric, composed of IFNAR1 and IFNAR2 for the type I IFN receptor and IFNλR1 and IL-10RB for IFNλ receptor. Receptor expression patterns differ with the IFNλR primarily localized to mucosal epithelial barrier sites and several immune cell populations including neutrophils, plasmacytoid dendritic cells (DCs), and macrophages, while type I IFN receptors are ubiquitously expressed (6, 7).

Differences between mouse and human IFNλ, as well as IFNλ subtype receptor affinities, add an additional layer of complexities when comparing studies. Humans produce IFNλ1–3 and occasionally IFNλ4, with the IFNλ1 dominant, while mice only produce IFNλ2–3, where IFNλ3 has dominant activity (8). As a result of these species-specific induction patterns, many studies regarding humans or human cells rely on IFNλ1, while murine studies use IFNλ3 or a mixture of IFNλ2 and 3. Because numerous studies show that all IFNλs are protective in both mouse and human respiratory viral infections, it is likely that their *in vivo* functions are similar. However, the analysis of the binding sites between each IFNλ and IFNλR1 showed that not all IFNλs are created equal. Miknis et al. identified that IFNλ1 possesses five cysteine residues to form disulfide bridges compared with seven found in both IFNλ2–3. However, the IFNλ residues directly binding to IFNλR1 are consistent among subtypes, and the biological activity of IFNλs has been described as IFNλ3>IFNλ1>IFNλ2 (9). It is possible that enhanced IFNλ3 antiviral activity could be caused by minor allele single-nucleotide polymorphisms (SNPs), which are known to cause increased responsiveness to influenza vaccination (10). However, one study showed that IFNλ2 and IFNλ3 can compensate for one another during murine pulmonary infections by selective deletion of IFNλ3 (11). Additional study of IFNλ subtypes is required to understand their common and distinct functions during infection to enhance the relevance of murine studies to the human population, as the use of IFNλ3 in mice may result in stronger phenotypes than what is seen with IFNλ1 in humans.

Because barrier sites, particularly the respiratory tract, have close contact with numerous foreign particles, activating immune responses that preserve the integrity of the barrier is important. IFNλ has uniquely been shown to clear respiratory viruses without inducing damaging inflammatory responses that type I IFN can cause, making it a popular candidate for new antiviral treatments (12 - 14). The benefits of IFNλ become more complicated, though, during non-viral respiratory infections, including bacterial and fungal infections and viral-induced super-infections. This review will outline the different roles of IFNλ that are context-, timing-, and infection-dependent.

## Distinct functions for IFNs

Differentiating the effects of IFNλ and type I IFNs (primarily IFNα/β) has been extensively reviewed by experts in the field (2, 8, 15). The following section will focus on distinguishing between these two types of IFNs with a narrower focus more relevant to the pulmonary infections discussed in this review. Although both type I IFNs and IFNλ signal through janus kinase/signal transducer and activator of transcription (Jak/STAT) pathways, resulting in the production of overlapping subsets of interferon-stimulated genes (ISGs), these interferons only have ~20% amino acid homology, resulting in the potential for non-redundant functions during infection (1). Studies show that often either type I IFN or IFNλ plays the dominant role during infection, but this can vary depending on the pathogen, infection site, and pathogen infection dose (16, 17).

Early data regarding IFNλ showed a tissue specificity not seen with type I IFNs (18 - 20). Compared with IFNα that induced ISGs in multiple tissue types and organs, IFNλ stimulation only induced ISGs in a small subset of tissues, primarily epithelial cells where expression of IFNλR1 is the highest (18). This represents a major difference between type I IFN and IFNλ, where type I IFN is able to act on any cell through its ubiquitously expressed receptor, but the activity of IFNλ is more restricted. Initial data showed that the heterodimeric IFNλR receptor, composed of IL-10RB and IFNLR1, was localized only on mucosal epithelial cells, but many other cell types have been identified as expressing the receptor, reviewed in Ref. (21), with the consequence of this expression being an active area of research.

Highly overlapping ISG subsets are induced by both type I IFN and IFNλ, with type I IFN inducing a greater number of genes than IFNλ (22). Specifically, only type I IFNs result in chemokine induction, including (chemokine (C-X-C motif) ligand) CXCL9, CXCL10, and CXCL11, through high activation of interferon regulatory factor 1 (IRF1), which is not seen during IFNλ signaling (23). Differences in ISG patterns may also be attributed to the partial reliance of IFNλ on MAPK (mitogen-activated protein kinase) signaling and activation, which type I IFNs do not require for optimal signaling (24). Although this study was performed in intestinal epithelial cells (IECs) and may not directly translate to the lung. A potential difference between IFNλ and type I IFNs is the kinetics of ISG induction: while type I IFNs cause an early and transient peak in ISGs after stimulation or viral challenge, IFNλ-induced ISGs were shown to have a delayed, but prolonged pattern of induction in a hepatocyte cell line (1, 23). Although data regarding lung-specific ISG induction in a controlled environment are limited, stimulation of human respiratory epithelial cells with IFNλ or IFNβ showed findings of dose-dependent ISG induction with early IRF1 activation seen only after IFNβ treatment, which is consistent with what is seen in hepatocytes (25). Clustering of IFNλ- and type I IFN-induced ISGs showed that both IFNs induce largely the same genes with distinct temporal patterns (24). IFNλ caused ISGs to peak more slowly than IFNβ, but concentrations of these genes continued to increase over time after IFNλ treatment rather than return to baseline (24). These distinctions are yet to be confirmed in the context of pulmonary infection.

Differences in production between IFNλ and type I IFNs are likely the cause of their disparate effects during infection. For instance, IFNλ is thought to be more protective against respiratory infections because it is produced early during infection and at higher concentrations than IFNα (1, 26). However, these effects seem to be virus-specific, as IFNα was shown to be protective against three major respiratory viruses, respiratory syncytial virus (RSV), rhinovirus (RV), and influenza virus, while IFNλ only protected against RV with partial effects against RSV (27). Similar results were shown using vesicular stomatitis virus, where cells required higher doses and treatment lengths of IFNλ to result in similar levels of antiviral activity compared with IFNβ (24). However, IFNs do not always act in the same manner. Mouse hepatitis virus (MHV) primarily causes induction of IFNλ over IFNβ in mice, but interestingly, loss of only IFNβ signaling and not IFNλ signaling caused increased susceptibility to infection (28). In this case, the importance of IFNλ during MHV infection was only elucidated using a very low- or high-dose infection. Even in this context, IFNλ had tissue-specific activity, promoting clearance in only the lungs while IFNβ increased MHV clearance in multiple organs (28).

These functional distinctions between IFNλ and type I IFNs show how they can alter the course of infection at a broad level. While the signaling pathways are similar, studies have already shown that this is not indicative of redundant functions. The following sections will discuss the roles of IFNλ on respiratory viruses, bacteria, and fungi in more detail, including when pathogens come together to induce a pulmonary super-infection; while no direct comparisons will be made to type I IFNs, other reviews have outlined the roles of type I IFNs during respiratory infections in detail (29 - 31). While IFNλ during viral infections has been well-studied, its functions during bacterial and fungal infections and super-infections are current areas of focus.

## IFNλ versus respiratory viruses

With IFNλ primarily acting at mucosal barrier sites, IFNλ-induced ISGs promote respiratory virus infection clearance and resolution. The following section will largely focus on influenza and severe acute respiratory syndrome coronavirus 2 (SARS-CoV-2) due to their global public health burden, but all respiratory viruses induce IFNλ production, sometimes preferentially, as part of activating the host innate immune response (17). Human metapneumovirus (hMPV), RSV, and RV all induce IFNλ and can cause infections that vary widely in severity (32 - 34). Interestingly, prophylactic treatment of mice with IFNλ promotes hMPV clearance while IFNλR1 expression correlates with more severe RSV and RV infections in human infants, suggesting that the role of IFNλ during pulmonary infections may be context- and species-dependent, which is explored in more detail elsewhere (32, 33, 35). Differences in the influence of IFNλ during these common pulmonary infections may also be model-specific, as a treatment before infection, while a common proof-of-concept approach in mice is not feasible in the human population.

### Influenza virus

IFNλ has been shown to play beneficial roles in many aspects of influenza infection, including initial responses, bridging innate and adaptive immune responses, and enhancing memory and vaccination efficacy. IFNλ production during viral infections is largely due to peroxisome-associated mitochondrial antiviral signaling proteins, and in the case of influenza, is mainly produced by epithelial cells both in the lung airways and in the alveoli (36 - 38). Infected cells are the primary producers of IFNλ, and this activity is dependent on interferon regulatory factors (IRFs), particularly IRF3 and IRF7 whose binding sites are prevalent in *ifnl* promoter regions (39). IFN signaling can also occur in a paracrine manner, which was shown to be important within the context of influenza infection. Ramos et al. (40) showed that levels of ISGs were lower in influenza-infected cells compared with bystander cells, and widespread production of IFNλ1 by both infected and uninfected cells promotes a broad antiviral state to prevent bystander cells from becoming infected. This activity of IFNλ1 was unique, as both type I IFNs and IFNλ2–4 are predominantly produced by influenza-infected cells (40).

**Fig 1:**
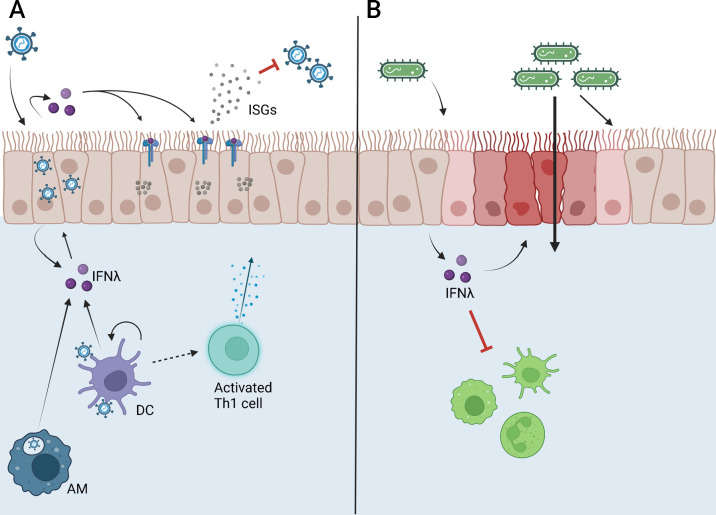
Functions of IFNλ during respiratory viral and bacterial infections. (**A**) IFNλ is produced by many cell types after viruses are recognized, including infected epithelial cells, activated macrophages (AMs), and dendritic cells (DCs). IFNλ can act in an autocrine or paracrine manner to induce ISG production that inhibits viral replication and transmission, limiting infection and enhancing clearance. IFNλ produced by DCs is required for the optimal activation of CD4^+^ Th1 cells and virus-specific CD8^+^ T cells. (**B)** Bacterial infection in the airways can induce IFNλ production primarily by epithelial cells, which can limit immune cell recruitment to the lung and decrease barrier integrity, leading to increased dissemination without impacting clearance of the bacteria from the lung. Figure created on Biorender.com.

Multiple additional studies have shown that IFNλ restricts influenza infection in mice. During influenza infection, IFNλs are produced by epithelial and immune cells, such as DCs, during both high- and low-dose viral infections and are found at much higher concentrations in the airways compared with type I IFNs (Fig. 1A) (1, 12). IFNλ primarily restricts viral replication early during infection to prevent the spread and excessive inflammation (12, 41). Epithelial cells and DCs both express the restricted chain of the IFNλ receptor, IFNλR1, as well as murine neutrophils, which are found near infected cells in influenza-infected mice (12). IFNλR1 knockout mice have been shown to have worsened infection in multiple studies, with increased viral spread and transmission as well as enhanced type I IFN production and neutrophilia, which can contribute to immunopathology (12, 41).

IFNλ can also be given therapeutically after influenza infection in mice to enhance infection resolution. Enhanced type I IFN and neutrophil responses in IFNλR1 knockout mice are ameliorated by giving mice pegylated-IFNλ (peg-IFNλ) (12). Peg-IFNλ given 1–2 days post-infection (dpi) resulted in lower viral titers and reduced immune cell infiltration into the airways at 7 dpi, as well as reduced lung pathology (12). This protective effect has been seen in multiple studies, with another paper showing IFNλ-mediated inhibition of viral replication without excessive inflammation when given before influenza infection or shortly afterward (13). Prophylactically, IFNλ2 prevented detectable viral replication, while treatment starting 2 dpi reduced the burden 10-fold (13). IFNλ induces antiviral gene expression rather than pro-inflammatory genes, resulting in lower cytokine production from immune cells and lower levels of epithelial apoptosis, resulting in a potent antiviral response without damaging inflammation, as seen in both mouse and human cells (13). Additional reports using human alveolar macrophages (hAMs) and primary influenza-infected human lung slices have shown that IFNλ is induced in hAMs after influenza infection and its signaling is required for clearance, as pre-treatment of these cells with IFNλ1 inhibits influenza infection experimentally (42).

With increasing data showing that IFNλ has therapeutic potential, the question then becomes: can IFNλ be re-purposed to enhance defenses against influenza virus before infection even occurs? These defenses include the antiviral Oseltamivir, commonly used after a positive diagnosis, and the annual influenza vaccine, which is the most important prevention mechanism against serious illness. In a study where mice were vaccinated three times using gamma-irradiated influenza vaccine alone or with IFNλ3 as an adjuvant, adding IFNλ3 to the vaccine enhanced both humoral- and cell-mediated immune responses against influenza infection 1 week after the final vaccination. The adjuvanted vaccine led to higher levels of total IgG as well as preferential production of IgG2a, skewing toward a Th1 (T helper cells) response compared with the non-adjuvanted vaccine (10). Vaccination with IFNλ3 also increased splenocyte proliferation after antigen restimulation compared with vaccination alone, with increased production of both Th1 and Th2 cytokines. The addition of an IFNλ3 adjuvant increases survival after a lethal influenza challenge (80% compared with 50% survival with irradiated virus alone [10]).

Similar suggestive results were seen in humans by studying healthy volunteers and the known IFNλ SNPs that can alter the production and function of multiple IFNλs (43). Patients with the less common IFNλ3 minor allele SNPs (TG or GG compared with major allele genotype TT) have increased seroconversion after influenza vaccination and enhanced protection specifically against two antigens, with alterations seen in both activated influenza-specific T cells and human leukocyte antigen (HLA)-expressing B cells (43). IFNλ also has an active role in the adaptive immune response against the influenza virus in mice. IFNλ signaling in CD103^+^ DCs, which express IFNλR1, was required for the formation of effective influenza-specific CD8^+^ T cells (44). IFNλR1 knockout mice showed decreased levels of CD8^+^ T cells and antigen-presenting cell migration to draining lymph nodes through day nine of infection, resulting in decreased survival of these mice compared with wild-type during a heterosubtypic influenza challenge (44).

Finally, IFNλ has also been shown to improve the efficacy of Oseltamivir (Tamiflu) by prolonging the emergence of viral strains that are resistant to antivirals in *in vitro* cell culture models. Tamiflu is relatively effective at preventing viral replication and spread, but a single amino acid change in viral neuraminidase is enough to confer resistance (45). IFNλ can be given in combination with Tamiflu starting at passage 1 to synergize treatment effects. This combination treatment was shown to decrease the emergence of resistant strains in cell culture from passage 6, seen in Tamiflu treatment alone, to passage 12 in the presence of increasing Tamiflu and IFNλ concentrations (45).

The breadth of research into the roles of IFNλ during influenza infection shows its protective effects during both innate and adaptive responses and its potential to prevent serious infection altogether. IFNλ promotes antiviral responses both as a therapeutic and an adjuvant in multiple different models, outlining its versatility and protective qualities.

### SARS-CoV-2

Compared with IFN responses during influenza infection, innate immune activation in response to SARS-CoV-2 infection is much more complex. SARS-CoV-2 is a novel member of the Coronaviridae family that also includes severe acute respiratory syndrome coronavirus 1 (SARS-CoV-1), Middle East respiratory syndrome coronavirus (MERS-CoV), and common cold viruses. SARS-CoV-2 has a lower fatality rate than SARS-CoV-1 or MERS-CoV at ~10% and ~34%, respectively, but has resulted in much higher total deaths due to its increased transmissibility among humans (46).

IFNλ induction during the onset of SARS-CoV-2 correlates with infection outcomes. Shahbazi et al. showed that SARS-CoV-2 patients admitted to the intensive care unit (ICU) had levels of IFNλ1 and IFNλ2 that were comparable to baseline healthy controls while non-ICU SARS-CoV-2 patients had significantly elevated levels of both IFNλ1 and IFNλ2. In another comparison between patients who recovered from SARS-CoV-2 infection and those who did not, IFNλ1 levels were significantly higher in individuals who recovered from infection (47). However, the kinetics of IFNλ induction can vary widely during SARS-CoV-2 infection, making the relationship between IFNλ and viral clearance more complex.

The timing of IFNλ production seems to play a role in whether it will be beneficial or detrimental to the host, with early production during SARS-CoV-2 infection correlating with worsened infection (48). SARS-CoV-2 often does not induce any IFN production, but IFNλ is upregulated more so than type I IFNs. Individuals with robust IFNλ induction at early time points after infection positively correlated both with those who became critically ill and with a length of hospital stay. Paradoxically, once an individual was admitted to the ICU, higher IFNλ levels were related to lower viral loads and faster clearance after hospital admittance (48). Another study collected tissue samples from children with permissive or non-permissive SARS-CoV-2 infections and found that cells from non-permissive infections produce IFNλ faster after *in vitro* re-infection than cells from permissive infections (49).

Although the interplay between IFNλ and SARS-CoV-2 can be complex, once again attention turned to the potential of IFNλ as a therapeutic, especially after such experimental success against the influenza virus. IFNλ inhibits SARS-CoV-2 replication in multiple *in vitro* systems when given prophylactically, including primary human airway epithelial cells and immortalized human epithelial cell lines (49, 50). Compared with influenza virus inhibition, restriction of SARS-CoV-2 by IFNλ seemed to be less robust but present nonetheless (50). Interestingly, IFNλ1 only exerted antiviral activity *in vitro* against SARS-CoV-2 at doses of 100–500 ng/mL when given before the viral infection and not as a therapeutic post-infection, while IFNλ1 given pre- or post-infection at 100 ng/mL showed restriction of influenza replication (50). Clinical trials for therapeutic peg-IFNλ report a therapeutic effect, with peg-IFNλ inducing a more rapid decrease of SARS-CoV-2 RNA over time, comparing 80% of patients clearing the virus 7 dpi to 63% of controls (14). Another trial showed that early treatment with peg-IFNλ also significantly decreased the rates of hospitalization or emergency department visits in patients with SARS-CoV-2 compared with placebo controls in addition to again resulting in faster viral clearance (51). Further analyses of these patient samples revealed elevated ISGs in IFNλR-expressing cells, including plasmacytoid DCs and B cells, after treatment without impacting activation and expansion of SARS-CoV-2-specific antibody and T cells (52).

Mouse models largely confirm what was seen in human cell culture models. IFNλ was shown to be protective against both upper- and lower-respiratory tract infections with SARS-CoV-2 in multiple mouse models, including conventional (inbred laboratory) mice and human angiotensin-converting enzyme 2 (hACE2) transgenic mice, while IFNλR1 knockout mice showed increased viral titers 5–7 dpi (53). Additionally, IFNλ2 given prophylactically (16 hours before infection) or therapeutically (1-2 dpi) protects mice from multiple SARS-CoV-2 strains at 3 dpi, including the beta and omicron variants that have spread rapidly on their emergence (53).

Another distinction between murine and human studies is the timing of treatment in non-clinical studies, which will impact the feasibility of using IFNλ as a therapeutic. Multiple reports show that IFNλ administration improves the clearance of multiple viruses in mice and humans, supporting clinical trials for influenza or SARS-CoV-2 treatment (12 - 14). However, most studies in mice involve IFNλ treatments that are prophylactic or early during the infection course where humans would not yet be diagnosed. When early treatments are possible in humans, as seen in the cited clinical trials, IFNλ has potent antiviral activity, but strong endogenous IFNλ production early during SARS-CoV-2 infection has also been linked to increased disease severity (43, 46). IFNλ treatments given later during viral infections, after viral clearance when tissue repair is prominent, have not been well-studied in humans, but IFNλ given to mice late during infection has been shown to impair repair and resolution, discussed in sections below. It is possible that IFNλ becomes less effective over time because IFNλR1-expressing epithelial cells become necrotic and less responsive to IFNλ; it has been established that influenza exposes new binding sites on epithelial cells during infection, and this could include the loss of IFNλR1 (54). Support for early administration of IFNλ is also seen in its ability to enhance virus-specific T-cell responses, where treatment after DC migration to draining lymph nodes may not be effective (37). Further study into the timing of IFNλ administration after infection, specifically those relevant to when humans would be diagnosed and seeking treatment, will identify the potential breadth of IFNλ efficacy in humans.

In addition to the consideration of treatment timing, potential side effects need always be considered, especially when cytokines are introduced exogenously to the body at potentially high levels. This becomes especially relevant when taking into consideration viral-induced super-infection, seen commonly with influenza and bacteria including *Staphylococcus aureus* and *Streptococcus. pneumoniae*. New data suggest that bacterial infections both alone and in a recovering lung can induce IFNλ which influences infection outcome.

## Ambiguous role of IFNλ with bacterial infections

Compared with the wealth of research concerning IFNλ during respiratory virus infections, much less is known about the roles IFNλ may play during bacterial infections, specifically those known to cause pneumonia. Bacterial pneumonia can have many causative agents, including Gram-positive bacteria like *S.aureus*, *S. pneumoniae*, and *Listeria monocytogenes*, or Gram-negative bacteria such as *Klebsiella pneumoniae*, *Pseudomonas aeruginosa* (PA), *Legionella pneumophila*, or *Bordatella pertussis* (55). Cell culture models have shown that a bacterial challenge results in IFNλ induction in a variety of cell types, including primary monocyte-derived DCs and human epithelial cell lines, leading to strengthened epithelial cell barriers (56 - 58).

### Gram-positive bacterium

IFN induction during *S. aureus* infection has been more extensively studied with type I IFNs than IFNλs, but Peignier et al. (59) showed that induction levels of both IFNβ and IFNλ vary widely by clinical isolates without any obvious striations between methicillin-resistant (MRSA) and methicillin-sensitive strains (59). While multiple studies have shown that *S. aureus* infection results in increased IFNλ levels in the lung, there is not a consistent conclusion as to whether IFNλ is helpful or harmful in clearing bacteria (60, 61). One study showed that global IFNλR1 knockout mice have reduced bacterial burden in the airways at 4 and 18 hours post-MRSA (strain USA300) infection and in the lungs 18 hours after infection (60). Another study confirmed these findings and further showed that depletion of the IFNλR led to enhanced clearance in both the bronchoalveolar lavage (BAL) fluid and lung tissue by 24 hours post-infection, with decreases in pro-inflammatory cytokines that usually lead to lung damage (62). Of note, IL-1β production was largely inhibited in IFNλR1 knockout mice, which was shown to be caused by reductions in neutrophil-specific pro-IL-1β processing (62).

*In vitro* data also showed that pre-treatment of healthy human nasal epithelial cells with IFNλ1 results in enhanced antibacterial activity against another MRSA strain, RN6390, although these results were not confirmed in airway epithelial cells (61). IFNλ1 was also shown to increase bacterial uptake and killing in differentiated human macrophage THP-1 cells, which is an interesting deviation from data showing faster clearance in IFNλR1 knockout mice.

One potential explanation for the discrepancies between the above studies could be the ability of *S. aureus* to survive as an intra- or extra-cellular pathogen (63). Although it is not a common respiratory pathogen, IFNλ2 was shown to have a positive influence on clearance of the intracellular Gram-positive bacterium *L. monocytogenes* (64). Pathogenic *L. monocytogenes* strains secrete a protein, LntA, which can interact with chromatin repressor bromo adjacent homology domain containing 1 (BAHD1) to modulate IFNλ and subsequent ISG expression (64). IFNλ is the most abundant IFN produced after *L. monocytogenes* infection, and *L. monocytogenes* strains where LntA is constitutively active have increased IFNλ and ISG expression, leading to faster clearance of bacteria (64). The authors of this work conclude that selective LntA is beneficial for the bacteria to prevent alerting the host immune response to the infection, which could also be an explanation for IFNλ increasing *S. aureus* clearance in cell culture models. Additionally, clearance of bacteria from the nasal tissue and upper airways may not accurately reflect mechanisms in the lower airways and alveoli, as the upper airways represent the likely first site of infection.

The paucity of data regarding the interplay of IFNλ and Gram-positive respiratory pathogens makes it difficult to draw clear conclusions, especially considering distinctions between *in vitro* versus *in vivo* and upper versus lower airways, but mouse models show that IFNλ may have negative impacts on bacterial clearance and infection resolution.

### Gram-negative bacterium

Similarly, IFNλ has been shown to have a context-dependent role during multiple Gram-negative bacterial lung infections. Many Gram-negative bacterial infections seem to be exacerbated by IFNλ through multiple mechanisms (60, 65 - 67). IFNλ is induced after *B. pertussis*, *K. pneumoniae*, and PA infections and kinetics vary by infection: for example, IFNλ peaks at 4 hour post-infection during PA infection and peaks between 4 and 10 days after *B. pertussis* infection (60, 65, 66). Regardless of the kinetics and intensity of IFN responses, these infections are ameliorated when IFNλ is disrupted. IFNλR1 knockout mice show reductions in lung bacterial loads at 18 hours after PA infection, while IFNAR knockout mice do not show the same results, indicating that IFNλ alone contributes to exacerbations (60). Interestingly, another study showed that when IFNλ2 was administered with PA and 8 hours post-infection, mice had decreased weight loss and immune cell infiltration into alveoli without reduction in bacterial loads, indicating that in this context IFNλ2 may decrease lung inflammation and pathology without increasing clearance (67).

IFNλ has been implicated in altering barrier function and inflammation in *B. pertussis* and *K. pneumoniae* infections as well. Both IFNλR1 knockout mice and IFNλR1/IFNAR double knockout mice infected with *B. pertussis* had reduced lung pathology scores compared to wild-type and IFNAR single knockout mice, suggesting that IFNλ was involved in driving pathology without impacting bacterial clearance (Fig. 1B) (66). Along the same lines, IFNλ was shown to increase barrier permeability in human airway epithelial cells and alter genes relating to barrier integrity in mice during *K. pneumoniae* infection. IFNλR1 knockout mice were not only protected from bacteremia compared to wild-type mice but also had significantly faster bacterial clearance in the lungs and the airways 4 dpi (65).

While the functions of IFNλ during lung bacterial infections are still active areas of research, we begin to see a context-dependent role emerging where IFNλ may be harmful or helpful based on the infecting bacteria and experimental conditions. This becomes especially relevant when bacterial infections are complicated by a viral infection, aptly named super-infections.

## IFNλ in super-infections

The term super-infection is used to describe the event of a primary viral infection being followed closely temporally by a secondary bacterial infection. During super-infections, the respiratory immune environment has not yet returned to a steady state, altering the antibacterial immune responses normally generated. Influenza-MRSA and RSV-PA super-infections are among the most common, and numerous reviews outlining super-infection immune responses exist (54, 68 - 71). Because super-infections involve both a virus, where IFNλ is beneficial, and a bacterium, where the role of IFNλ is more questionable, these models provide more insight into the temporal and contextual roles of IFNλ than a single infection model.

Many factors can contribute to super-infection susceptibility and onset, including barrier permeability and subsequent repair during influenza infection and resolution. Studies have shown that lung epithelial cell damage and lack of tissue repair are prominent explanations for increased morbidity and mortality after super-infection compared to single influenza or bacterial infection (72, 73). During viral infections, alveolar type 2 cells quickly proliferate 5–7 dpi to promote barrier repair and protection after viral clearance, but this response is altered during secondary bacterial complications (74). At this time, IFNλ is the predominant IFN present in the lungs and has been shown by two groups to delay epithelial cell proliferation after a viral challenge (74, 75). Chronic IFNλ exposure prevented bacterial clearance over time and IFNλ stimulation in super-infected mice reduced both epithelial cell proliferation, by inducing genes in the p53 pathway, and differentiation into specialized cell types (74). Conversely, IFNλR1 knockout mice had increased cell proliferation after a viral challenge with fewer red blood cells and immune cells in BAL fluid with increased survival after the secondary *S. pneumoniae* challenge (74).

While multiple studies have shown that IFNλ increases the severity of super-infection, the specific mechanism by which this occurs is still being elucidated (11, 74, 76, 77). IFNλ treatment during super-infection was shown to reduce neutrophil recruitment to the airways and neutrophil phagocytosis of both *S. pneumoniae* and *S. aureus*. Further, IFNλ levels in BAL fluid of super-infected mice correlate positively with the bacterial burden (11, 76). However, the influence of IFNλ on antimicrobial peptide (AMP) production is controversial: studies have been published showing no effect on AMP production after an IFNλ treatment and that IFNλR1 knockout mice have increased levels of several AMPs, including regenerating family member III gamma and neutrophil gelatinase associated lipocalin (76, 77). During influenza infection, IFNλ strongly induced the ISG, indoleamine-2,3-dioxygenase (IDO), which has been shown to suppress innate immune responses (78). Blocking IDO modulates viral titer, immune cell recruitment, and T-cell activation, and under wild-type conditions may have an indirect role in increasing super-infection severity (78, 79).

Super-infection with other pathogens, including PA and RSV or RV, also has interplay with IFNs where the primary infection can modulate a secondary infection. Studies with PA super-infection often use the bacteria as the primary infection to be consistent with what is commonly seen in patients with cystic fibrosis (CF). Primary PA infection in CF cells causes a defect in IFN and ISG production after viral infection, resulting in impaired clearance via increased viral spread rather than replication (80, 81). PA genes LasR, which controls LasA and LasB proteins, and AprA were required for the degradation of IFNλ protein, with little to no detection of IFNλ as early as 14 hours post-RSV infection, but isolates from chronic PA infections lost protease expression over time (81).

**Fig 2:**
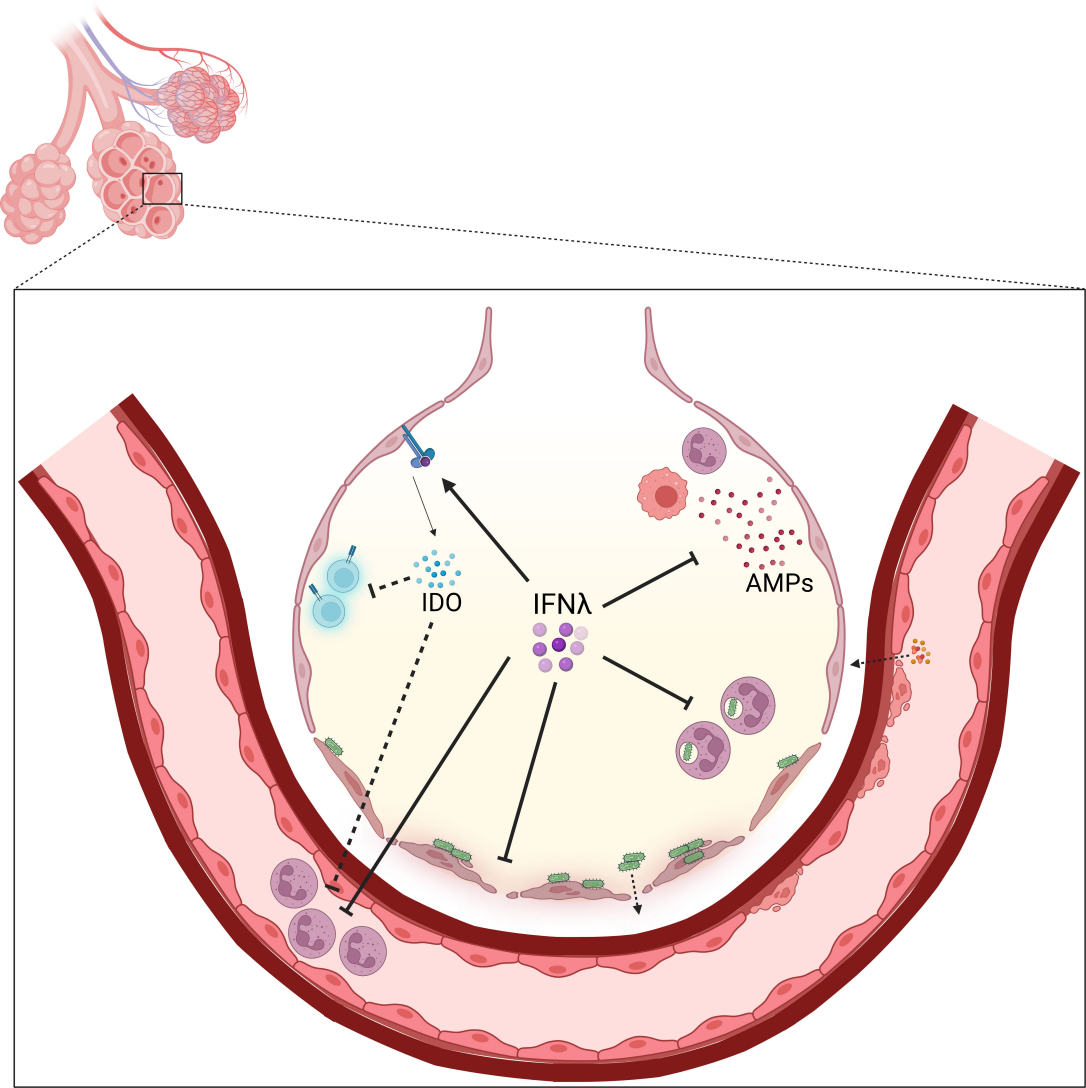
Mechanisms of IFNλ-dependent bacterial exacerbation in the post-virally infected lung. Representative alveolus in the lung during the recovery period after respiratory viral clearance. IFNλ presence in this timeframe has many roles that can act in tandem and parallel to exacerbate secondary bacterial infection, including suppressing antibacterial AMPs, neutrophil recruitment to the lung and phagocytosis of bacteria, and delaying barrier restoration and integrity. IFNλ can also act indirectly by promoting ISGs, including IDO, that have secondary functions of inhibiting T-cell activity and immune cell recruitment during super-infection. Figure created on Biorender.com.

The outcome of IFNλ signaling seems to be largely context-dependent, even within super-infection. While IFNλ is beneficial against viral infections, the continued presence of IFNλ past a certain optimal timeframe results in an impaired ability to fight secondary bacterial infections (Fig. 2). The use of IFNλR1 knockout mice illustrates this phenotype, where primary viral infections are not yet lethal, but mice are better able to control bacterial super-infection (74, 76, 77). Interestingly, the positive impacts of IFNλ can still be seen in PA-RSV super-infections, where lack of IFNλ resulted in decreased clearance of the secondary virus (80, 81).

## Emerging: IFNλ in fungal infections

In addition to bacterial single and super-infections, a more comprehensive profile for IFNλ comes into view as new studies illuminate the antifungal capacity of IFNλ. Extensive work from the Rivera group has shown potent antifungal roles for IFNλ during *Aspergillus fumigatus* infection. IFNλ produced by CD45^+^ cells was required for optimal reactive oxygen species (ROS) production and neutrophil extracellular traps (NETs) to clear the fungus, with IFNλR1 knockout mice showing impaired antifungal activity and increased fungal burden in the lungs (82). More recent work has shown that this activity of IFNλ is dectin-1-dependent, which is the host receptor that recognizes *A. fumigatus* and induces IFNλ production. Dectin-1 knockout mice showed increased susceptibility to a fungal infection that could be reversed by administering recombinant IFNλ (83). *A. fumigatus* can also be involved in pulmonary super-infection, where primary influenza infection increases the secondary fungal burden and causes more severe disease (84). While IFNλ has yet to be specifically studied in this model, STAT1 signaling during super-infections was shown to impair antifungal neutrophil activity via decreased CXCL1 and CXCL2, leading to increased fungal burden (84). Because STAT1 is one of the major signaling proteins involved in the IFNλ pathway, it is possible that IFNλ may play similar roles during both viral–bacterial and viral–fungal super-infection.

## Unanswered questions in the field

Although IFNλs are the most recently discovered family of IFNs, there are almost two decades worth of expansive research into their many roles and functions, which is only continuing to grow. IFNλ has clear antiviral activity against respiratory viruses, including influenza and SARS-CoV-2, which currently represent two of the highest causes of death annually. Importantly, IFNλ can be distinguished from type I IFNs during infection, as they have distinct induction kinetics and half-lives and induce overlapping sets of ISGs that have unique temporal expression patterns (24). IFNλ is also much less inflammatory than type I IFNs without any discernible differences in antiviral potency, which led to increased popularity as a potential antiviral treatment and successful clinical trials where peg-IFNλ led to faster SARS-CoV-2 clearance (12 - 14).

However, the idea that IFNs, especially IFNλs, are only involved in viral infections is constantly shifting as new evidence emerges showing the interplay between IFNλ and other pathogens. Data show a more controversial role for IFNλ during pulmonary bacterial infections and super-infection, where the lung is in an active recovery phase after viral clearance. Studies have come to multiple conclusions regarding the functionality of IFNλ against bacteria: evidence has shown that IFNλ both enhances and inhibits clearance and increases dissemination of bacteria from the lung without impacting localized clearance (60, 61, 65, 66). These conclusions seem to vary among species, but some organisms like *S. aureus* do not have a consistent phenotype. The addition of a primary viral infection complicates the system further, where levels of IFNλ are already elevated in the lung due to the virus. IFNλ directly and indirectly contributes to exacerbated super-infections by inhibiting immune cell recruitment and antibacterial activity and prolonging barrier repair and inducing inhibitory ISGs, respectively (74 - 78 - 78). Specific functions of IFNλ have yet to be elucidated in other models of pulmonary super-infection, including that of influenza and *A. fumigatus*, but similar to influenza, IFNλ has shown to be beneficial in the clearance of fungus.

Other factors that may influence IFNλ induction and activity have also emerged in recent years, including crosstalk between IFNλ and the commensal microbiome. The influence of the microbiome on IFNλ is particularly intriguing given the restriction of IFNλR1 expression largely to mucosal barrier sites, where the colonization of commensal microbes is common. Commensal microbes have known roles in enhancing or inhibiting IFN responses against pathogens, and epithelial cells lining both the oral mucosa and intestinal tract preferentially produce IFNλ over type I IFN (85, 86). Chronic inflammation, which occurs with periodontitis, can lead to decreases in IFNλ production and activity after viral challenge. This was shown to be exacerbated in particular by commensal bacteria *Porphyromonas gingivalis*, which is also associated with periodontitis (85). *P. gingivalis* colonization inhibited IFNλ production after viral infection by suppressing IRF1 and STAT1 activation and transcriptionally repressing IFNλ1 production via zinc finger E-box binding homeobox 1 (ZEB1) (85). In the gut, the microbiome provides tonic pattern recognition receptor (PRR) stimulation to IECs, leading to IFNλ production and IFNλ-induced homeostatic ISGs that were protective against rotavirus infection in mice (86). These homeostatic ISGs were produced by mature enterocytes rather than cells in the crypts and were linked to the microbiome and IFNλ specifically by using broad-spectrum antibiotics and IFNλR1 knockout mice, both of which caused ablation of homeostatic ISGs (86). Little is known about the role of IFNλ in regulating the microbiome in the nose or lungs.

This recent and ongoing work shows the functional relevance of IFNλ beyond viral infections, and these new emerging roles are still being discovered. IFNλ is a member of the IL-10 cytokine family due to its use of the IL-10RB chain as one-half of its receptor (87). In addition to its similarities with type I IFNs, comparisons to other IL-10 family members, namely IL-22 have started to be identified. Antibacterial roles of IL-22 are well-characterized (88 - 90), and several studies have identified regulation of IFNλ by IL-22 and vice versa during pulmonary bacterial infections (65, 67). These new avenues of potential regulation of and by IFNλ are an exciting area of continuing research, particularly as nuances of IFNλ in bacterial and fungal infections are still being uncovered.

Identifying these context-dependent roles for IFNλ will be critical for determining the practicality of its use as an antiviral treatment. While IFNλ strongly inhibits viral replication and protects the host from excessive damage and inflammation, current data show that this protective effect is not consistent for all pulmonary infections. Influenza and other respiratory viruses are known to prime the lung for secondary bacterial and fungal infections (84, 89), and high levels of IFNλ in the lung at this time have already been shown to slow tissue repair and super-infection recovery (74, 77). IFNλ as a therapeutic would need to balance between its beneficial antiviral qualities and its detrimental function against clearing infection sequelae. Specific mechanisms of IFNλ-mediated resolution or infection prolongation are still being identified in super-infection, which will be key to fully understand its role in pulmonary infection.
